# PVP-Assisted Solvothermal Synthesis of High-Yielded Bi_2_Te_3_ Hexagonal Nanoplates: Application in Passively Q-Switched Fiber Laser

**DOI:** 10.1038/srep15868

**Published:** 2015-10-29

**Authors:** Xin He, Hang Zhang, Wei Lin, Rongfei Wei, Jianrong Qiu, Mei Zhang, Bin Hu

**Affiliations:** 1 School of Applied Physics and Materials, Wuyi University, Jiangmen 529020, China; 2 State Key Laboratory of Luminescent Materials and Devices and Institute of Optical Communication Materials, South China University of Technology, Guangzhou 510640, China; 3 Wuhan National Laboratory for Optoelectronics (WNLO), Huazhong University of Science and Technology (HUST), Wuhan 430074, China

## Abstract

High-yielded Bi_2_Te_3_ hexagonal nanoplates were fabricated via a facile solvothermal method with the assistance of poly (vinyl pyrrolidone) (PVP). Effects of PVP molecular weight and concentration on the morphology and size distribution of the products were illustrated in this study. Molecular weight of PVP is significant for determining the morphology of Bi_2_Te_3_. The hexagonal nanoplates with high yield were obtained in the presence of PVP with molecular weight of 40000–45000. The average size and size distribution of Bi_2_Te_3_ nanoplates can be slightly varied by controlling concentration of PVP. High-yielded Bi_2_Te_3_ nanoplates exhibit characteristics of saturable absorption, identified by open-aperture Z-scan technique. The synthesized Bi_2_Te_3_ nanoplates with large saturation intensity of 4.6 GW/cm^2^ and high modulation depth of 45.95% generated a stable passively Q-switched fiber laser pulse at 1.5 μm. In comparison with recently reported Q-switched fiber lasers utilizing exfoliated Bi_2_Te_3_ nanosheets, our passive Q-switching operations could be conducted at a relatively low threshold power of 30.2 mW or a quite high output power of 99.45 mW by tuning the cavity parameters.

Second generation topological insulator (TI) materials such as Bi_2_Te_3_, Bi_2_Se_3_ and Sb_2_Te_3_ with common structural characteristics of gapped insulating bulks and metallic conducting surfaces have gained great attention in recent years^1^,^2^,^3^,^4^,^5^. TI surfaces exhibit the linear Dirac spectrum dispersion, resulting in a unique electronic property^6^; however, optical properties and applications require further exploration. Since Bernard *et al.* initially identified Bi_2_Te_3_ as an effective saturable absorber in 2012^7^; the application values of TIs in nonlinear photonics have received enhanced research interests. Wen and Zhang’s group has worked to promote the development of TIs-based nonlinear photonics. They made researches to explore the applications of TIs-based saturable absorbers in mode-locking and Q-switching from optical to microwave region^8^,^9^,^10^,^11^,^12^,^13^,^14^,^15^,^16^,^17^. Lee *et al.* utilized bulk-structured Bi_2_Te_3_ with modulation depth of 7.7% to generate passively Q-switched 1.89 μm fiber laser with a threshold of 175 mW and maximum output power of 0.68 mW^18^. Chen *et al.* synthesized layered Bi_2_Te_3_ with a liquid exfoliation approach, and the modulation depth and saturation intensity of the layered Bi_2_Te_3_ was ~22% and ~57 MW/cm^2^ at 1.5 μm, respectively. The stable passively Q-switched operation utilizing this obtained Bi_2_Te_3_ could be performed at a threshold of 67 mW. The maximum output power and pulse energy is 19.56 mW and 1.525 μJ, respectivley^19^.

It is well know that the following condition needs to be satisfied to obtain stable continuous-wave mode locking without Q-switching instabilities^20^.


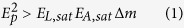


where *E*_*p*_ is the intracavity pulse energy, and Δ*m* is the modulation depth of the saturable absorber. *E*_*L,sat*_ and *E*_*A,sat*_ represent the effective saturation energy of the gain medium and saturable absorber, respectively. In order to generate stable Q-switched pulses, the saturable absorber requires a large saturation energy and high modulation depth to suppress mode-locked pulses from fiber laser with the cavity dispersion well controlled.

However, the saturation energy and modulation depth are largely determined by the morphology and size of the saturable absorber. Bulk-structured TIs or exfoliated TI flakes generally exhibit large size/thickness or irregular profile with planar dimensions of several micrometers. In this case, a large saturation energy and high modulation depth are hardly obtained due to the increased absorption cross-section of the ground state and decreased metallic surface states^21^. Moreover, the poor dispersion of the bulk-structured TIs or exfoliated TI flakes in the solvents might degrade the performance repeatability/stability of optoelectronic devices.

Therefore, fabrication of the nanostructured-TIs with high yield is expected to generate a large saturation energy and high modulation depth, and to obtain stable Q-switched pulses. In our work, TI: Bi_2_Te_3_ hexagonal nanoplates were synthesized via a solvothermal process. The nanoplates retain average size controlled from 650.8 to 736.5 nm, thickness of approximately 10 nm, and uniform size distribution. Excellent dispersibility in various solvents of the nanoplates is also obtained. The nanoplates exhibit the saturable absorption characteristic, identified by open-aperture Z-scan measurement. Stable passively Q-switched operations were performed at 1.5 μm utilizing the screened nanoplates with uniform size distribution due to the high modulation depth of 45.95% and large saturation intensity of 4.6 GW/cm^2^.

Consequently, a systematic investigation was conducted from the controllable synthesis of Bi_2_Te_3_ hexagonal nanoplates to the application in passively Q-switched laser at 1.5 μm. The saturable absorber with nanostructure was revealed to be significant in promoting the performance of Q-switched lasers and the nanostructured Bi_2_Te_3_ nanoplates are also expected to be successfully applied in performance improvement of the other optoelectronics devices.

## Experimental

Bi_2_Te_3_ hexagonal nanoplates were synthesized by a modified solvothermal process according to the references^22^,^23^. Typically, 1 mmol bismuth chloride, 1.5 mmol sodium tellurite, 8 mmol sodium hydroxide and PVP with certain qualities was weighted. BiCl_3_, Na_2_TeO_3_ and NaOH were respectively dissolved in 10 mL ethylene glycol (EG) with ultrasound applied to obtain transparent solutions. PVP was added to EG solution of BiCl_3_ and then magnetically stirred until completely dissolved (designated as S1). EG solution of Na_2_TeO_3_ was then introduced into S1 to generate a milky solution. Once the NaOH added, the mixed solution became transparent again and the homogenous solution was transferred to a sealed 50 mL Teflon autoclave and heated at 180 °C for 36 h. The resulting products displayed a gray-black color and were washed repeatedly with ethanol by centrifugations.

X-ray diffraction (XRD) patterns were characterized by a Bruker diffractometer utilizing Cu Kα radiation (λ = 0.15418 nm). Product dispersed in ethanol was deposited on glass slide surface and XRD data collected from the dried coatings. Raman spectra were recorded on a Raman spectrometer (Renishaw inVia, Gloucestershire, UK) utilizing a 532 nm laser as the excitation source. Morphologies and sizes of the products were observed by a field-emission scanning electron microscope (FESEM, Nova NanoSEM430) and a transmission electron microscope (TEM, JEOL-2100F). High-resolution TEM (HRTEM) and selected area electron diffraction (SAED) were conducted on the JEOL-2100F to characterize the microstructure of the nanoplates, and the surface topology and thickness of Bi_2_Te_3_ nanoplates were determined by an atomic force microscope (AFM, Nanoscope IIIa, Veeco) with a tapping mode.

## Results and Discussion

### Effects of PVP molecular weight and concentration on the morphology and size distribution of Bi_2_Te_3_ nanoplates

Morphologies of Bi_2_Te_3_ products significantly are dependent on several experimental factors in the solvothermal process such as reaction temperature, concentration of alkali and surfactant type^24^,^25^,^26^; however, PVP molecular weight and concentration on the formation of Bi_2_Te_3_ nanoplates is rarely discussed. Yield and size distribution of Bi_2_Te_3_ hexagonal nanoplates as related to PVP is further researched in this study.

**Figure 1:**
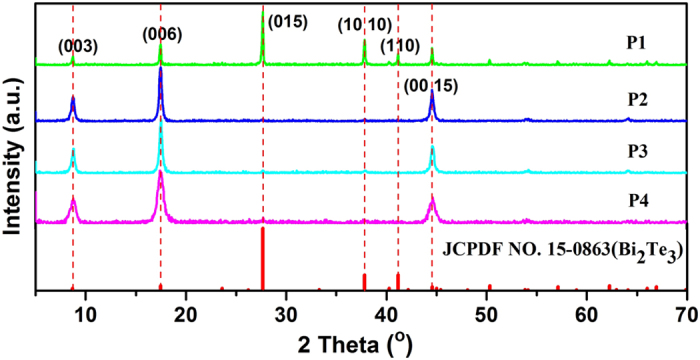
XRD patterns of the product P1, P2, P3 and P4; the standard PDF card of Bi_2_Te_3_ (JCPDF NO. 15–0863) was provided for reference.

The effect of PVP molecular weight on the growth of Bi_2_Te_3_ was examined as the products were synthesized in the presence of PVP with molecular weights of 10000, 40000, 45000 and 58000 (named as P1, P2, P3 and P4), respectively. Figure 1 illustrates XRD patterns of the product P1-P4 and the standard card of Bi_2_Te_3_ (JCPDS File NO. 15–0863 from ASTM). Comparing the patterns of products with the standard PDF card of Bi_2_Te_3_, the detected diffraction peaks from each product can be well indexed to the standard Bi_2_Te_3_ rhombohedral structure. High phase purity of the prepared Bi_2_Te_3_ was indicated as no other crystalline impurities were observed. Obvious differences in relative peak intensity among four patterns exist, demonstrating that Bi_2_Te_3_ from four products exhibit distinguishing characteristic of the preferred orientation growth.

The strongest diffraction peak occurs at 27.7^o^ in the standard PDF card of Bi_2_Te_3_, corresponding to (015) plane. Relative intensity of the diffraction peak at 17.4^o^ assigned to (006) plane is greatly enhanced as the molecular weight of PVP increases. The peak area ratios of (006) to (015) plane are 0.57, 30.13, 26.35 and 23.12 for the product P1-P4, respectively. The result suggests that four products present preferred orientation growth of Bi_2_Te_3_ along c-axis by various degrees with sample P2 exhibiting the most intense orientation growth.

**Figure 2:**
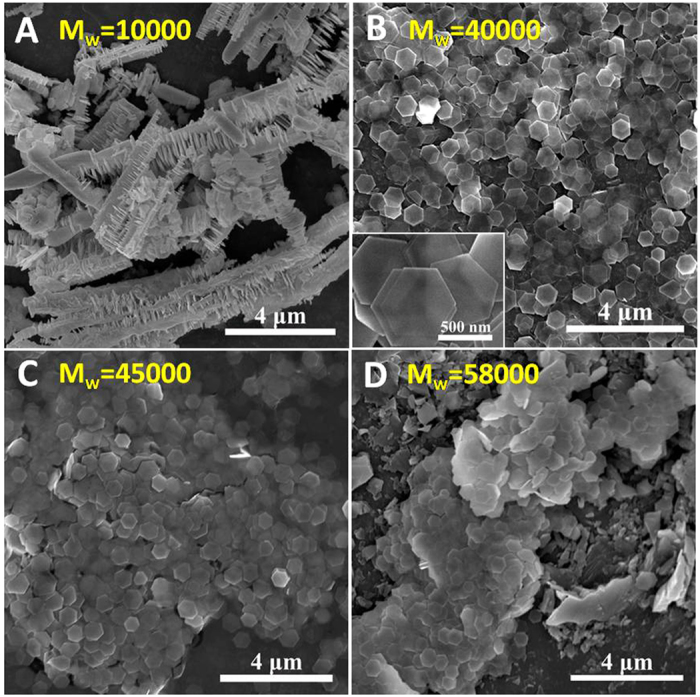
SEM images of the product P1 (A), P2 (B), P3 (C) and P4 (D), respectively; the inset of (B) is a magnified SEM image of the representative Bi_2_Te_3_ nanoplates.

Figure 2A–D illustrates SEM images of the product P1-P4. Bi_2_Te_3_ hierarchical structures were prepared with PVP molecular weight of 10000, as indicated in Fig. 2A. Figure. 2B shows Bi_2_Te_3_ hexagonal nanoplates with high yield were obtained when the molecular weight of PVP was increased to 40000. The inset of Fig. 2B presents a magnified SEM image of the representative hexagonal nanoplates with distinct edges and corners. Figure 2C demonstrates that high-yielded hexagonal nanoplates were also achieved as molecular weight of PVP reached 45000. The product P3 displays a poorer monodispersity of the nanoplates than the product P2 providing explanation for the products P2 and P3 sharing similar high yield of the nanoplates but different peak area ratio of (006) plane to (015) plane. In the XRD measurement, monodispersed nanoplates tended to lie on the surface of the glass slides and exhibited intense growth oriented along the c-axis. The product P3 shares a similar yield of the nanoplates as the product P2, yet the quantity of nanoplates lying on the glass slides is relatively less due to poor monodispersity and the assembly of the nanoplates, leading to the decrease in the peak area ratio of (006) to (015) plane for the sample P3. The yield of Bi_2_Te_3_ nanoplates declined and irregular morphology bulk was detected in the product P4 as molecular weight of PVP further increased to 58000, as shown in Fig. 2D.

Molecular weight of PVP is significant for determining the morphology of Bi_2_Te_3_. Formation of Bi_2_Te_3_ with various morphologies in the presence of PVP was explored through a proposed mechanism. TeO_3_^2-^ and Bi^3+^ may react with EG with the help of NaOH, as the reacting rate of TeO_3_^2-^ is faster than that of Bi^3+^^27^, Te is initially formed. Subsequently, the reduced Bi compounds with Te to form Bi_2_Te_3_ in the presence of PVP. PVP is a type of surfactant with the repeated units and the molecular weight is determined by the repeated numbers. When PVP with small molecular weight was added, the collision probability among Te atoms in the solution was relatively high. Te atoms would preferentially form into rod-like Te prior to Bi^3+^ were reduction^28^. The reduced Bi atoms compounded with rod-like Te then tend to form Bi_2_Te_3_ with a hierarchical structure. Molecular weight of PVP improves with the increase of the repeated unit numbers, leading to the decrease of collision probability among Te atoms and providing increased potential for latish compound of Te with Bi. Bi_2_Te_3_ nanoplates with high yield were obtained in this case. As molecular weight of PVP was further raised, the viscosity of solution was found to exceed the ability to obtain a homogeneous reaction and the yield of Bi_2_Te_3_ nanoplates decreased.

**Figure 3:**
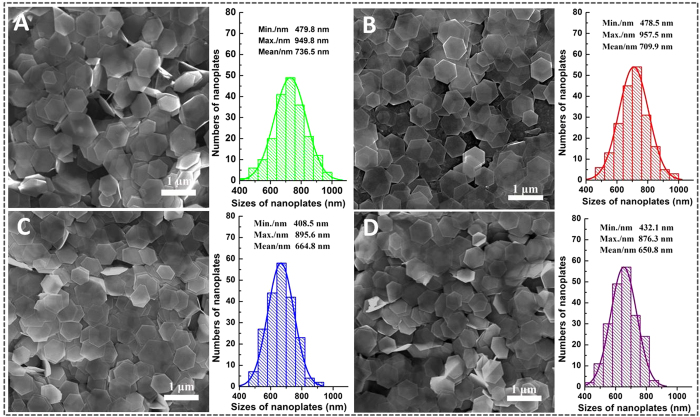
SEM images and size distribution histograms of the synthesized Bi_2_Te_3_ hexagonal nanoplates in the presence of 5.55 (A), 11.1 (B), 2.2 (C) and 44.4 μmol (D) PVP with the molecular weight of 40000.

The effect of PVP concentration on the growth of Bi_2_Te_3_ nanoplates was examined as products were synthesized in the presence of 5.55, 11.1, 22.2 and 44.4 μmol PVP with molecular weight of 40000 (named as Q1, Q2, Q3 and Q4), respectively. Figure 3A–D displays SEM images and size distribution histograms of these four products. Bi_2_Te_3_ hexagonal nanoplates with high yield were obtained in all products. To further evaluate the average size and size distribution of Bi_2_Te_3_ hexagonal nanoplates in four products, the sizes of approximately 200 nanoplates were measured in each product and the average size of the nanoplates was calculated. Size was defined by the diagonal length of hexagonal nanoplates (as illustrated in Fig. S1) and average size of the Bi_2_Te_3_ nanoplates was gradually decreased from 736.5 to 650.8 nm with the addition of PVP from 5.55 to 44.4 μmol.

The standard deviation (SD) and standard error (SE) of the nanoplates’ size for the product Q1, Q2, Q3 and Q4 were calculated to evaluate size distribution of the nanoplates, respectively (The formulas are presented in the supporting information). Calculated values of the SD, SE and SE% for four products are summarized in Table 1.

The values of SD, SE and SE% varied in a small range though the average size of Bi_2_Te_3_ hexagonal nanoplates gradually decreased with the addition of PVP increased. Among four samples, the minimum value of SD, SE and SE% was 82.73 nm, 5.85 nm and 0.899%, respectively, obtained from the sample Q4 prepared with 44.4 μmol PVP. Further increase in the concentration of PVP caused the value of SD, SE and SE% for the nanoplates’ size to increase tremendously to 142.63 nm, 9.34 nm and 1.330%, potentially as a result of the inhomogeneous reaction in a largely viscous solution (SEM image and size distribution histogram of the product prepared with 66.6 μmol PVP is presented in Fig. S2).

**Figure 4:**
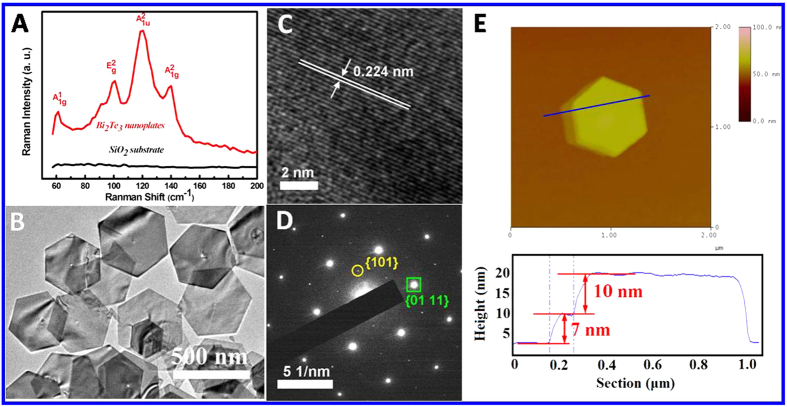
(**A**) Raman spectra of Bi_2_Te_3_ nanoplates and SiO_2_ substrate; (**B**) TEM image of Bi_2_Te_3_ hexagonal nanoplates; (**C**) HRTEM image and (**D**) SAED pattern of one nanoplate. (**E**) AFM topography image and the height profile of Bi_2_Te_3_ nanoplates.

The microstructure of the nanoplates with uniform size distribution from the sample Q4 was further characterized by Raman scattering spectrum, as indicated in Fig. 4A. Spectrum of SiO_2_ substrate was provided for reference as the spectrum was collected from the sample deposited on the SiO_2_ substrate. Four Raman peaks at 61.1, 100.8, 119.7 and 139.7 cm^−1^, stemmed from 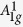, 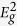, 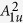 and 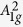 of Bi_2_Te_3_ nanoplates, respectively. Appearance of 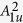 at 119.7 cm^−1^ is due to the crystal symmetry breaking of the nanoplates and is absent in the Raman spectrum of bulk-structured Bi_2_Te_3_ as a result of inversion symmetry^29^. The peak of 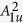 exhibits the highest relative intensity in Raman spectrum in this study, revealing nanoplate thicknesses are likely exceptionally thin. Figure 4B presents a TEM image of the representative hexagonal nanoplates with flat surfaces and sharp edges. The ripple-like contrasts are derived from the microstrain. Figure 4C displays a HRTEM image taken from the edge area of one selected nanoplate, presenting the continuous lattice fringes with a plane spacing of 0.224 nm, which corresponds to the (01 11) lattice planes of rhombohedral Bi_2_Te_3_ (JCPDS File NO. 15–0863 from ASTM). The SAED pattern of the nanoplate indicates the single crystalline nature of Bi_2_Te_3_, as shown in Fig. 4D. The sets of spots marked with circle and rectangle are indexed to the {101} and {01 11} planes of rhombohedral Bi_2_Te_3_ phase, respectively. Figure 4E displays an AFM topography image and height profile of two stacked Bi_2_Te_3_ nanoplates, revealing that the nanoplates retain flat surfaces and thicknesses of approximately 10 nm, which is in good agreement with the result of the Raman spectrum.

Bi_2_Te_3_ hexagonal nanoplates with high yield were controllably synthesized using a PVP-assisted solvothermal method and the sample Q4 with uniform size distribution was screened to further characterize the nonlinear optical properties.

### Generation of Q-switched fiber laser pulses based on the saturable absorber of high-yielded Bi_2_Te_3_ nanoplates

**Figure 5:**
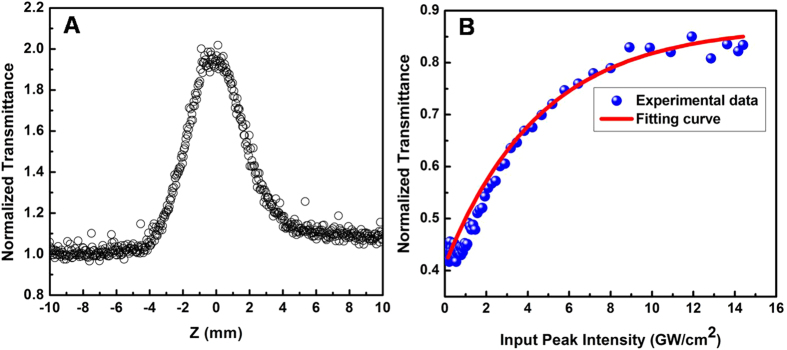
(**A**) Open-aperture Z-scan trace; (**B**) Transmission characteristics of high-yielded Bi_2_Te_3_ nanoplates under 800 nm irradiation.

Figure 5 A presents a typical trace of open-aperture Z-scan measurement for the Bi_2_Te_3_ nanoplates. A sharp peak located at the beam focus can be observed, confirming the nonlinear saturable absorption characteristic of the Bi_2_Te_3_ nanoplates (A schematic diagram of the Z-scan experimental setup is presented in Fig. S3). Variation of the normalized transmittance with the input peak intensity is depicted in Fig. 5B. We fitted the data utilizing the equation (2)^30^,^31^;


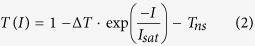


where, *T*(*I*) and Δ*T* are the transmittance and modulation depth; *I* represents the input pulse intensity of 14.4 GW/cm^2^; *I*_*sat*_ is the saturation intensity and *T*_*ns*_ is the non-saturable loss. The obtained values of Δ*T*, *I*_*sat*_ and *T*_*ns*_ are 45.95%, 4.6 GW/cm^2^ and 12.98%, respectively.

**Figure 6:**
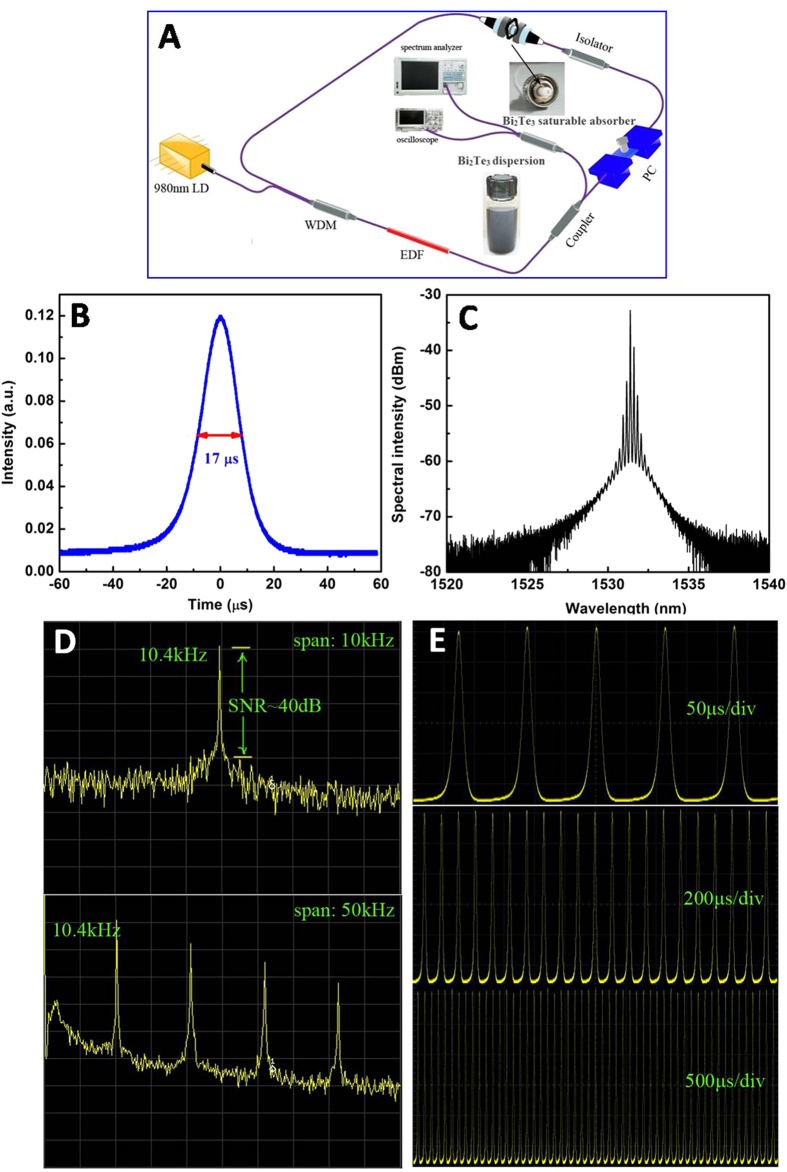
Typical Q-switching pulse emitted from fiber laser using high-yielded Bi_2_Te_3_ nanoplates as a saturable absorber at the pump power of 33.0 mW. (**A**) Schematic diagram of a passively Q-switched fiber laser; (**B**) Single Q-switching pulse profile; (**C**) Output spectrum of Q-switching pulse; (**D**) Radio-frequency optical spectra at the 10.4 kHz with the span of 10 and 50 kHz, respectively; (**E**) Various oscilloscope traces with different ranges of Q-switching pulse.

Therefore, the high-yielded Bi_2_Te_3_ nanoplates were explored, in this study, as a saturable absorber in passive Q-switch erbium-doped fiber laser (EDFL) to generate laser pulses, due to the high modulation depth of 45.95% and the large saturation intensity of 4.6 GW/cm^2^ 32,33,34,35. Figure 6A describes the schematic diagram of a passively Q-switched EDFL based saturable absorber of Bi_2_Te_3_ nanoplates, incorporated with polymethyl methacrylate (PMMA) to fabricate a flexible Bi_2_Te_3_/PMMA film for characterization. The total cavity length is approximately 12 m, mainly consisting of a 2 m-long Er-doped gain fiber and 10 m-long SMF-28e as the pigtails of the corresponding components. A 980/1550 nm wavelength division multiplexer (WDM) was employed to act as a coupler in the pump laser and the laser output beam was extracted by a 10% output port of a 90:10 fiber coupler. A polarization controller (PC) was originally incorporated for optimization of the birefringence in the laser system, while a polarization independent isolator was inserted into the cavity for the unidirectional beam oscillation. The Bi_2_Te_3_/PMMA composite film was placed between two fiber connectors and integrated into the laser cavity for the Q-switching operation.

Stable Q-switched pulses were achieved at a relatively low threshold pump power of 30.2 mW and proven to be indifferent to the adjustment of PC. Figure 6B provides a single pulse profile at a pump power of 33.0 mW. The Q-switching single pulse with a repetition rate of 8.1 kHz and output power of 68.5 μW was stable with no significant pulse jitter observed on the oscilloscope and a symmetric intensity profile was exhibited with pulse duration of 17 μs. Typical optical spectrum of the Q-switching pulse is depicted in Fig. 6C, revealing a central wavelength of the pulse at 1531 nm. The etalon effect produces a multipeak structure in the optical spectrum as a result of reflection light interference from two fiber connectors’ end. Observation of such fine structure is not possible without the insertion of an integrated saturable absorber device.

RF output spectrum of Q-switching pulses was measured at the same pump power of 33.0 mW to investigate laser stability. Figure 6D illustrates the RF spectra with a span of 10 and 50 kHz, respectively. The repetition rate of the pulse train is 10.4 kHz and signal-to-noise ratio (SNR) is approximately 40 dB. Stability of the Q-switching operation is confirmed in this study as no other frequency component, except the fundamental and harmonic frequency, was observed in the RF spectrum even in a wider span of 50 kHz. The Q-switched pulse is in a stable operating state as illustrated by the oscilloscope traces at different ranges with no significant pulse modulation (Fig. 6E), suggesting this Q-switching operates in a widely stable regime.

**Figure 7:**
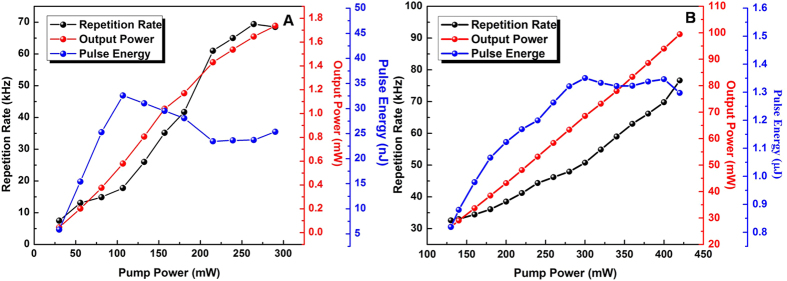
Variation of the repetition rate, the output average power and the pulse energy of the Q-switched pulses with the pump power. (**A**) The total cavity length is 12 m, and a 10% fiber coupler is applied to couple out the laser emission. (**B**) The total cavity length is 28 m, and a 50% fiber coupler is applied to couple out the laser emission.

Dependences of the repetition rate, output power and pulse energy on the pump power were indicated in Fig. 7A. The output power of the stable Q-switching lasers can be increased from 0.044 to 1.737 mW when the pump power is enhanced from 30.2 to 290.3 mW. The repetition rate simultaneously is varied in the range of 7.5–68.5 kHz. In this case, a low Q-switching threshold and a wide range of repetition rate can be obtained. However, the maximum output power and pulse energy are relatively low in comparison with recently Q-switched fiber lasers utilizing similar saturable absorber. The compared performance parameters are summarized in Table 2. In order to obtain a high energy from the Q-switched laser based on the high-yielded Bi_2_Te_3_ nanoplates, we changed the cavity parameters of the laser. The total cavity length was extended to approximately 28 m, and a 50% fiber coupler was applied to couple out the laser emission. Stable Q-switched laser pulses can be observed as the pump power is increased from 120 to 420 mW. The typical oscilloscope traces of the Q-switched pulses under the pump power of 140 mW and 380 mW were shown in Fig. S4, demonstrating that the Q-switching pulse output is stable at every specific repetition rate and pump power. The relationship between the repetition rate, output power and pulse energy of the Q-switched pulses with respect to the incident pump power is summarized in Fig. 7B. The output power and repetition rate increase almost linearly with the pump power. The repetition rate can be changed in the range of 32.6–76.7 kHz. The maximum output power is up to 99.45 mW under the pump power of 420 mW. The pulse energy can be varied between 0.88 μJ and 1.35 μJ. The maximum pulse energy of 1.35 μJ is obtained under the pump power of 300 mW. Our investigation suggests that the high-yielded Bi_2_Te_3_ hexagonal nanoplates can be one promising saturable absorber with a low Q-switching threshold or a large damage threshold for Q-switched fiber lasers in high-power operation.

## Conclusion

High-yielded Bi_2_Te_3_ hexagonal nanoplates were synthesized with the assistance of PVP via a solvothermal method. Morphology and size distribution of Bi_2_Te_3_ were dependent on the PVP molecular weight and concentration. The Bi_2_Te_3_ nanoplates with well dispersion and uniform size distribution exhibit saturable absorption characteristic, identified by Z-scan technique. Stable passively Q-switched laser pulses utilizing the Bi_2_Te_3_ nanoplates as a saturable absorber were successfully conducted in the 1.5 μm wavelength region, owing to the high modulation depth of 45.95% and the large saturation intensity of 4.6 GW/cm^2^. Compared to the exfoliated Bi_2_Te_3_ nanosheets, the high-yielded Bi_2_Te_3_ nanoplates can generate stable Q-switched pulses with a relatively low threshold power of 30.2 mW or a high output power of 99.45 mW through adjusting the cavity parameters of the laser.

## Additional Information

**How to cite this article**: He, X. *et al.* PVP-Assisted Solvothermal Synthesis of High-Yielded Bi2Te3 Hexagonal Nanoplates: Application in Passively Q-Switched Fiber Laser. *Sci. Rep.* **5**, 15868; doi: 10.1038/srep15868 (2015).

## Supplementary Material

Supplementary Information

## Figures and Tables

**Table 1:** The list of the average size, the calculated values of SD, SE and SE% for the nanoplates in the products Q1, Q2, Q3 and Q4, respectively.

Sample No.	n_PVP_/μmol	Average size/nm	SD/nm	SE/nm	SE%
Q1	5.55	736.5	103.56	7.32	0.994
Q2	11.1	709.1	96.07	6.79	0.957
Q3	22.2	664.8	104.06	7.36	1.107
Q4	44.4	650.8	82.73	5.85	0.899

**Table 2:** Output performance comparison of passively Q-switched fiber lasers using the exfoliated Bi_2_Te_3_ nanosheets and the high-yielded Bi_2_Te_3_ nanoplates.

Saturable Absorbing Materials	Wavelength (nm)	Q-Switching Threshold (mW)	Repetition Rate Range (kHz)	Max. Output Power (mW)	Max. Pulse Energy (nJ)	Refs
Exfoliated Bi_2_Te_3_ nanosheets	1543.2	45	9–12 (△_R_ = 3)	0.043	4	13
	1566.9	67	2.15–12.8 (△_R_ = 10.65)	19.56	1525	19
High-yielded Bi_2_Te_3_ nanoplates	1531	30.2	7.5–68 (△_R_ = 60.5)	1.74	32.6	This work
		120	32.6–76.7(△_R_ = 44.1)	99.45	1350	
