# Supplementary material for: PVP-Assisted Solvothermal Synthesis of High-Yielded Bi2Te3 Hexagonal Nanoplates: Application in Passively Q-Switched Fiber Laser
Source: Sci Rep. 2015 Oct 29;5:15868. doi: 10.1038/srep15868 (PMC4625368; doi:10.1038/srep15868)
Supplement: Supplementary Information [file srep15868-s1.doc]

**PVP-Assisted Solvothermal Synthesis of High-Yielded Bi_2_Te_3_ Hexagonal Nanoplates: Application in Passively Q-Switched Fiber Laser**

Xin He1,2[[1]](#footnote-2)*, Hang Zhang^2^, Wei Lin^2^, Rongfei Wei^2^, Jianrong Qiu2[[2]](#footnote-3)*, Mei Zhang^1^, Bin Hu^3^

*^1^School of Applied Physics and Materials, Wuyi University, Jiangmen 529020, China*

*^2^State Key Laboratory of Luminescent Materials and Devices and Institute of Optical Communication Materials, South China University of Technology, Guangzhou 510640, China*

*^3^Wuhan National Laboratory for Optoelectronics (WNLO), Huazhong University of Science and Technology (HUST), Wuhan 430074, China*

*
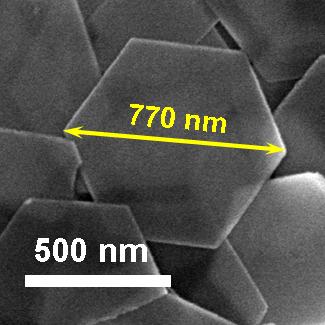
*

Fig. S1. SEM image of the representative Bi_2_Te_3_ nanoplates; the size was defined by diagonal length of the hexagonal nanoplates.

The values of SD and SE were determined by the formulas (1) and (2);

(1)

$SE=\frac{SD}{\sqrt{n}}$ (2)

The percentage of SE was calculated utilizing the formula (3);

$SE\%=\frac{SE}{R}\times100\%$ (3)

where, $R$ is the average size of Bi_2_Te_3_ nanoplates.


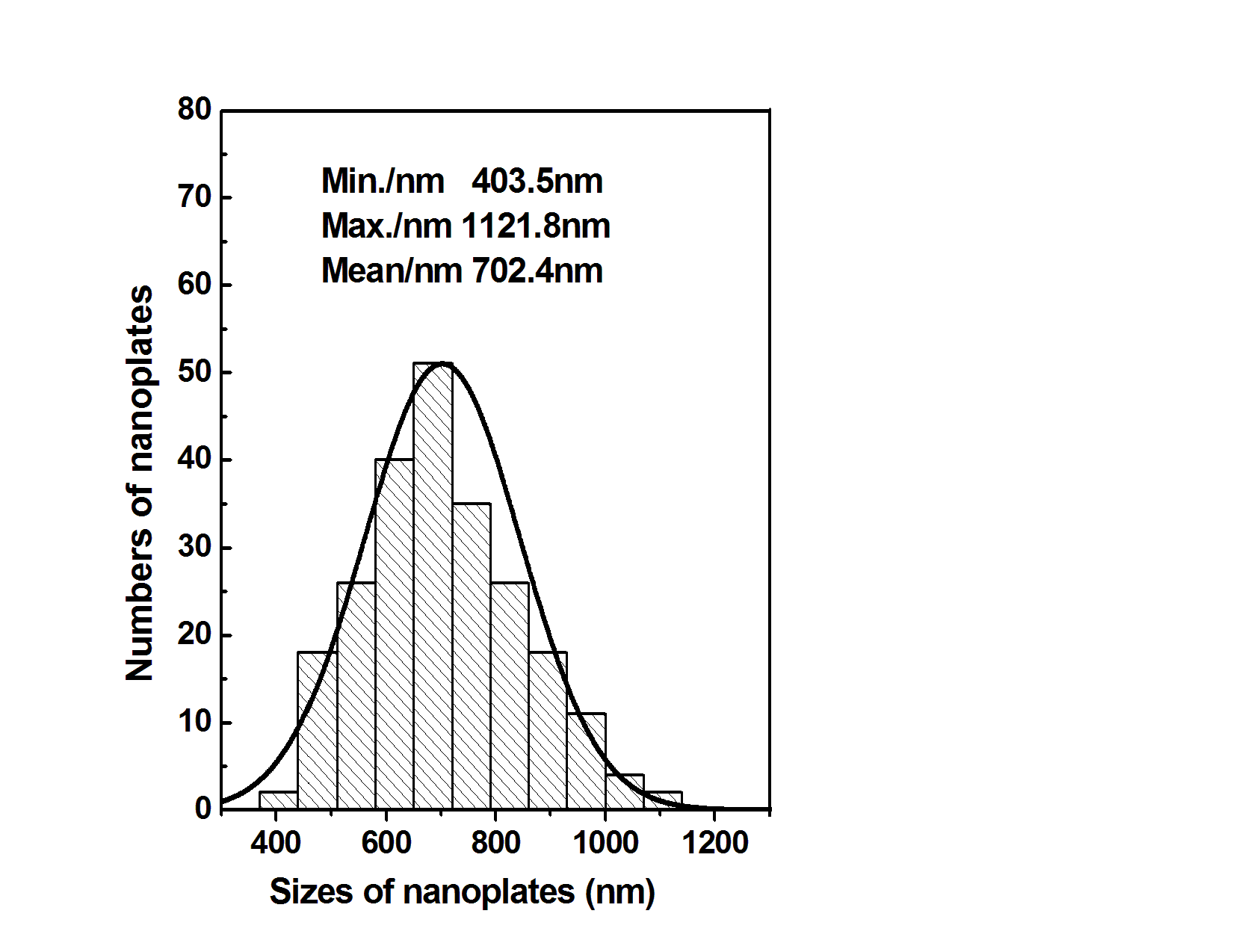

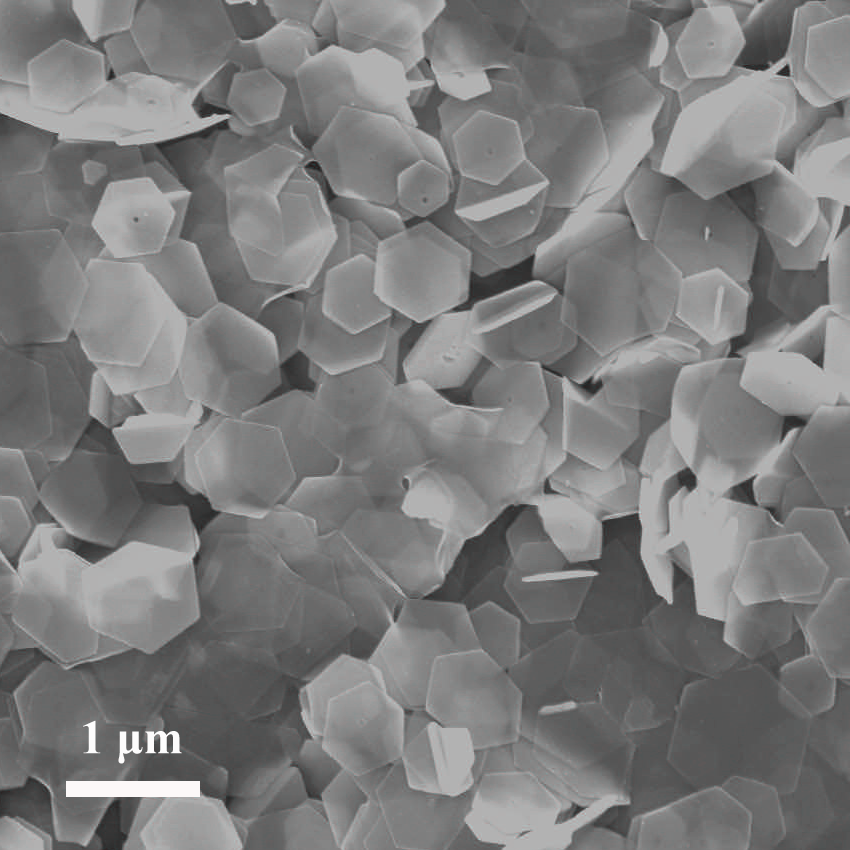


Fig. S2. SEM image and size distribution histogram of the product prepared with 66.6 μmol PVP; the calculated value of SD, SE and SE% is 142.63 nm, 9.34 nm and 1.330%, respectively.

Fig. S3. Schematic diagram of Z-scan experimental setup. The experiment was performed with a mode-locked Ti: sapphire laser that delivered laser pulse of 130 fs at 800 nm with a repetition rate of 1 kHz. An ethanol solution contained Bi_2_Te_3_ nanoplates was dropped onto a 1 mm thick quartz plate, and dried at room temperature to fabricate the sample for Z-scan characterization.


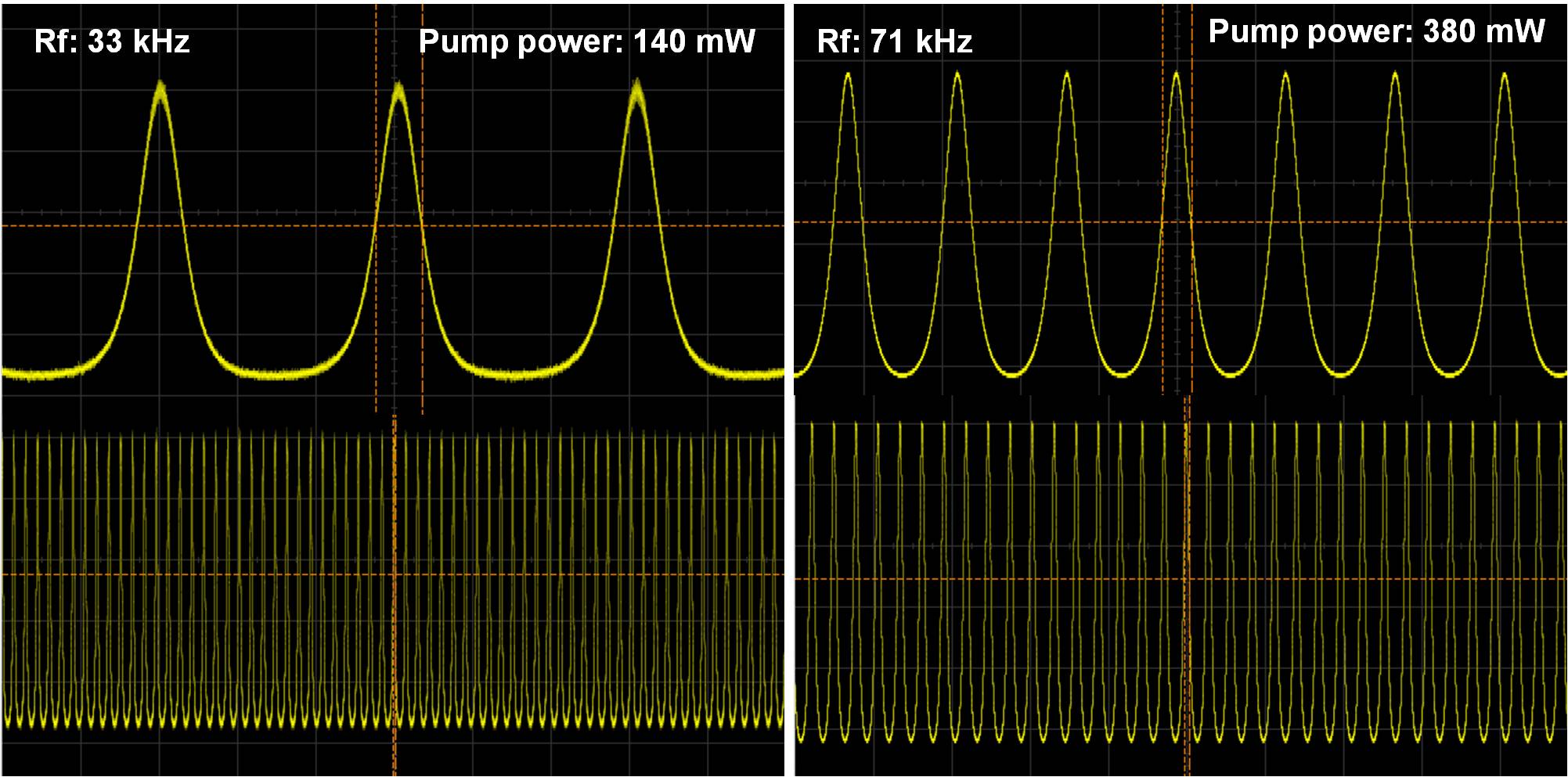


Fig. S4. The various pulse trains obtained under different pump powers.

1. ^^ Corresponding author. Tel. +86 750 3296040; Fax: +86 750 3296400.

   E-mail address: [hexinwyu@126.com](mailto:hexinwyu@126.com) (X. He);

   E-mail address: [qjr@scut.edu.cn](mailto:qjr@scut.edu.cn) (J. Qiu) [↑](#footnote-ref-2)
2. ^^ [↑](#footnote-ref-3)
